# TRAIT SELF-CONTROL AND ATTACHMENT ANXIETY IN RETIREES’ DEPRESSIVE SYMPTOMS AND EMOTIONAL WELL-BEING

**DOI:** 10.1093/geroni/igac059.2899

**Published:** 2022-12-20

**Authors:** Jensine Paoletti, E Lim Lydia Wu-Chung, Ryan Brown, Michelle Chen, Angie LeRoy, Christopher Fagundes

**Affiliations:** Rice University, Houston, Texas, United States; Rice University, Houston, Texas, United States; Rice University, Houston, Texas, United States; Northwestern University, Evanston, Illinois, United States; Baylor University, Waco, Texas, United States; Rice University, Houston, Texas, United States

## Abstract

Trait self-control (TSC) is a well-known predictor of well-being. TSC may reduce distraction from hedonistic pursuits and enable more effective goal-directed behavior. Attachment anxiety (AA) is a trait that predicts lower relationship quality and lower well-being; people high on AA are characterized by hyper-sensitivity, proximity-seeking and excessive rumination. In retirement, these traits may interact to predict wellbeing. Methods: 120 retired participants took part in data collection (M = 62.37 years old; SD = 10.07 years; 68% women, 70% white). Data collection included self-reported AA (Experience in Close Relationships), TSC (Brief Self-Control Scale), depressive symptoms (Center for Epidemiological Studies Depression Scale) and emotional well-being (RAND 36-Item Health Survey). We conducted hierarchical linear regressions and accounted for demographic and health-related covariates. Results: TSC was negatively related to depressive symptoms and positively related to emotional well-being. AA was positively related to depressive symptoms and negatively related to emotional well-being. We found an interaction predicting depressive symptoms such that participants with low TSC and high AA had the highest depressive symptoms (p = .036). This interaction was not significant for emotional well-being. Discussion: After retiring, maintaining a healthy social life may be more effortful, as there are fewer ‘built-in’ social interactions (i.e., with coworkers). Retirees with higher TSC may be able to maintain their social life more effectively than those with lower TSC. Similarly, people with lower AA may be able to obtain more enjoyment from their social lives. Together, lower AA and higher TSC may be protective mechanisms for depressive symptoms.

